# ASR gene family: a case of tandem-drive evolution

**DOI:** 10.3389/fmolb.2025.1456645

**Published:** 2025-06-13

**Authors:** Nicolle Louise Ferreira Barros, João Pedro Carmo Filgueiras, Thomaz Stumpf Trenz, Guilherme Weber, Andreia Carina Turchetto-Zolet, Marcia Margis-Pinheiro

**Affiliations:** ^1^ Programa de Pós-graduação em Genética e Biologia Molecular, Departamento de Genética, Instituto de Biociências, Universidade Federal do Rio Grande do Sul (UFRGS), Porto Alegre, Brazil; ^2^ Programa de Pós-graduação em Biologia Celular e Molecular, Centro de Biotecnologia, Instituto de Biociências, Universidade Federal do Rio Grande do Sul (UFRGS), Porto Alegre, Brazil

**Keywords:** gene family evolution, intrinsically disordered proteins, ASR proteins, DNA-binding domain, residues substitution

## Abstract

**Introduction:**

ABA, Stress, and Ripening (ASR) proteins are characterized by the presence of the ABA/WDS domain and are involved in plant development processes and tolerance to abiotic and biotic stresses. Despite their importance as transcription factors or molecular chaperones, a complete understanding of their biological roles is limited by a lack of information on their mechanisms of action, protein structure, and evolutionary relationships between family members. Our previous molecular dynamics simulation analysis of rice OsASR5 suggested that H^91^, R^92^, H^93^, and K^94^, are the main residues involved in the interaction with DNA, essential for the transcription factor activity of this protein. However, the presence and conservation of the DNA-binding domain among ASR family members remain unknown. Likewise, there is a lack of phylogenetic analyses evaluating the evolutionary history of ASR proteins across major taxonomic groups, outside just the *Solanum* species.

**Methods:**

To address these gaps, we conducted a phylogenetic study and protein sequence analyses to gain insights into the evolution of ASR genes in plants. We performed a genome-wide identification of ASR genes via HMMER, using the ABA/WDS domain, in 163 Archaeplastida genomes.

**Results and discussion:**

Our results reveal that the potential origin of the ASR gene occurred in the common ancestor of Streptophytes (Charophytes and Embryophytes). Moreover, our study identifies ASR genes in seedless plants. The evolutionary relationship between 465 ASR homologs, found in 76 species, was estimated through maximum likelihood analysis. The results reinforce the rapid and dynamic evolution of the ASR gene family, reflected by the low support in the deep nodes of the phylogeny and the great variation in the number of ASRs in the genomes evaluated, and in some cases their complete absence. As for diversification, tandem duplications seem to be the main mechanism involved. Regarding the conservation of residues in the domain, only two of the 78 are widely conserved, such as E^79^ and H^93^. By analyzing the three-dimensional model, we noticed the interaction between them and we hypothesize that they are essential for the stabilization of the domain during interaction with DNA.

## 1 Introduction

Whole-genome duplications and clusters of gene duplication events are evolutionary processes that contribute to the origin and expansion of proteins. The diversity of protein families upholds the complex metabolic pathways that plants activate in response to endogenous and exogenous cues. Considering this, the inference of phylogenetic relationships is crucial for processing genomic data since it offers comparative insights into the roles of these biomolecules through evolutionary distance and guides experimental designs. Besides, these analyses can contribute to the understanding of plant physiology, given that the fate of duplicated genes in association with the ecology of the species determines the common or lineage-specific biological traits (Lynch and Conery, 2000; Carretero-Paulet and Fares, 2012; Li et al., 2016). Reconstructing the evolutionary history of gene families helps identify paralogy and orthology relationships (Jakobson et al., 2024) and, therefore, the classification of nomenclatures and subgroups (Wang et al., 2018), providing valuable information about the enrichment (Castro et al., 2017), redundancy, and functional transition of gene family members through group establishment (Tang et al., 2013). It also contributes to the identification of active residues under different selective pressures.

ASR (ABA, Stress, and Ripening) are so-called plant-exclusive transcription factors (TF) and in addition to regulating gene expression, they are involved with chromatin remodeling and maintenance of the native conformation of proteins under stress (Çakir et al., 2003; Wang et al., 2018; Atassanov et al., 2022). This family is associated with developmental processes and stress response (Chen et al., 2011; Arenhart et al., 2013), although information on the mechanisms of action is limited. ASR proteins are known to localize in different subcellular compartments (Arenhart et al., 2013), but the signals promoting protein translocation, the mode of binding to their targets, and the conformational transition they undergo remain unclear. To date, only five ASR proteins have been characterized as intrinsically disordered (Goldgur et al., 2006; Dai et al., 2011; Hamdi et al., 2017; Barros et al., 2023), of which only one had the topology of the binding complex resolved (Barros et al., 2023).

The conformational plasticity of ASR imposes experimental constraints that can be resolved by *in silico* tools, which can predict the chemical environment of each residue and its interactions to ensure the structural switches between the free and ligand-associated modes. Considering this, our group previously showed that the rice ASR5 protein (OsASR5) is intrinsically disordered since it is enriched with charged residues such as E, H, and K while lacking hydrophobic ones such as L and I, and β and bulky conformation (Barros et al., 2023). The growing interest in intrinsically disordered regions is driven by their abundance in TF protein sequences and the alleged role in stabilizing and guiding the DNA-binding domain in the search for motif-containing sites (for details, see Brodsky et al., 2021). Additionally, these regions might assist the TF transitions between DNA groves, overall spatial accessibility to co-factors, and post-translational modifications (Már et al., 2023). Besides ASR5 the rice genome encodes for five additional ASR gene members (Frankel et al., 2006), compared to 24 members in wheat (Yoon et al., 2021), one in grape (Çakir et al., 2003), and none in *Arabidopsis thaliana*, for example, whose absence imposes yet another drawback on the functional analysis of ASR gene family. Although ASR proteins have been identified in many species of economic interest, detailed evolutionary relationships among them remain unexplored.

To address these gaps, our group previously proposed the *in silico* three-dimensional model of the rice ASR5 protein and the zinc-dependent DNA binding (Barros et al., 2023), based on the metal-binding (positions one–16) and DNA-binding (positions 87–94) domains of tomato ASR1 (Kalifa et al., 2004; Rom et al., 2006). Furthermore, we concluded that residues H^91^, R^92^, H^93^, and K^94^ of the DNA-binding site and M^1^, K^5^, and K^14^ of the putative metal-binding site of OsASR5, along with their flanking sites, are crucial for the stability of the complex with a target gene (Barros et al., 2023). Therefore, the present study aimed to understand the molecular evolution of the ASR gene family across the Archaeplastida species and evaluate the composition of ASR domains and their flanking residues. Our results suggest these proteins are not exclusive to land plants, as the founding gene appears to originate from Charophyta. Also, the phylogenetic analysis indicates that tandem duplications promote the rapid diversification of family members, resulting in two main clades formed by ancient and recent groups and unidentified ASR proteins outside these groups. The results produced here contribute to the identification of active residues among ASR proteins, offering insights into how diffuse the structural disorder between them is, and supporting hypotheses about novel ASR functions.

## 2 Materials and methods

### 2.1 Database and sequence retrieval

To explore the evolutionary history of the ASR gene family, we performed a genomic identification of these genes in 163 Archaeplastida species. Among these species, three are Rhodophytes, one Glaucophyta, one Prasinodermophyta, 19 Chlorophyceae, nine Charophytes, seven “Cryptogams”, five Gymnosperms, four “early-diverging Eudicots”, 63 Eudicotyledons, and 51 Monocotyledons (Supplementary Table S1). For this purpose, we used HMMER’s hmmsearch (Mistry et al., 2013), employing the PFAM domain present in ASR proteins, the ABA/WDS induced protein (PF02496), and an e-value < 1e^−5^ was used as a threshold. All predicted polypeptide sequences from the genomes were retrieved via Ensembl Plants (https://plants.ensembl.org), Phytozome v13 (https://phytozome-next.jgi.doe.gov), or Phycocosm (https://phycocosm.jgi.doe.gov). For Phytozome, we used only the dataset containing the primary transcripts. For Ensembl and Phycocosm, as this dataset is not available, the primary transcripts were filtered after hmmsearch. Sequences with less than 65% of the domain coverage were removed from further analysis.

### 2.2 Sequence alignments and phylogenetic analysis

Two datasets were used to estimate the evolutionary history of the ASR gene family in plants. The first one contained 465 sequences from 76 species (hereby called ‘Streptophyta dataset’). The second one included 490 sequences from 46 species, focusing on the Poaceae family (hereby called the ‘Monocotyledon dataset’). The Streptophyta dataset contains species representing different taxonomic groups, from charophyte algae to angiosperms, while the Monocotyledon dataset only included species of monocotyledons, mainly species from the Poaceae family. Charophyte and Bryophyte ASR were used as roots in the Monocotyledon dataset. Both datasets were aligned using MAFFT with the L-INS-I strategy (Katoh et al., 2017). The resulting alignments were manually curated and trimmed, retaining only the region corresponding to the ABA/WDS protein domain (78 amino acids in length). However, for the Streptophyta dataset, only 74 residues were kept in the final alignment, due to the low quality of the alignment in the last four residues. The curated alignments were then used to estimate the phylogenetic trees. The phylogenetic analysis was carried out with the maximum likelihood method using IQTree v2.2.6 (Minh et al., 2020). The trees were estimated using 10,000 UltraFast bootstrap replicates (Hoang et al., 2017). Additionally, the parameters -pers 0.2 and -nstop 500 were used. Both datasets were analyzed in triplicate, and the final tree was selected based on the best log-likelihood value. The substitution models were determined by ModelFinder (Kalyaanamoorthy et al., 2017) and automatically selected based on the best Bayesian Information Criterion (BIC) value. In the Streptophyta dataset, the selected model was LG + I + R5, while in the Monocotyledon dataset, the Q.plant + R5 was selected. For more confidence in the topology found, we also estimated the phylogeny using PhyML (Guindon et al., 2010), with a support calculated from 200 Transfer bootstrap replications (Lemoine et al., 2018). For the Streptophyta dataset, we also used the LG + I + R5 model, while for the Monocots, we used the second best model proposed by ModelFinder, JTT + R4, due to the absence of “Q.plant” models in PhyML.

### 2.3 Selection analyses

In the study of coding gene evolution, a widely used measure is the ω value. This parameter estimates the selective pressure acting on a gene, where values less than one indicate purifying selection, with values closer to zero reflecting stronger pressure to conserve residues. Conversely, an ω value greater than one indicates positive selection, and the further it deviates from one, the stronger the selection promoting residue diversification. Values equal to or very close to one are indicative of neutral evolution, meaning that variations in residues do not result in significant changes in protein function (Álvarez-Carretero et al., 2023). To estimate selective pressures in the ASR gene family, a subdataset from monocotyledons was created. Coding sequences were retrieved exclusively from Poaceae species, with Joinvillea ascendens as the outgroup. The sequences were aligned at the protein level using MAFFT with the L-INS-I strategy (Katoh et al., 2017). Subsequently, PAL2NAL (Suyama et al., 2006) was used to convert the protein multiple sequence alignment into a codon alignment. The alignment was manually curated to retain only the ABA/WDS domain. At this point, all identical sequences were removed to reduce redundancy and avoid bias due to the high number of polyploids. The phylogeny of this subdataset was estimated using IQTree, and finally, EasyCodeML (Gao et al., 2019) was employed to detect signals of positive selection in the ASR gene family using the site model strategy (Yang et al., 2000).

### 2.4 Analysis of the three-dimensional model of the ASR domain

We used MEME (Bailey et al., 2015) for identification of conserved motifs within ASR protein sequences, in both Streptophyta and Monocotyledon dataset, with the maximum number of motifs set to 15, with minimum a width of six and a maximum width of 80 amino acids. WebLogo web software (https://weblogo.berkeley.edu/logo.cgi) was used to build the ABA/WDS domain logo from the sequences used. One logo was generated for the alignment of the Streptophyta dataset and another for the Monocotyledon dataset. Furthermore, based on the phylogeny of the Monocotyledon dataset, a sequence logo was constructed for each set of sequences grouped into the clades of ASR1, ASR2, ASR3, ASR4, ASR5, and ASR6 to evaluate whether key amino acids were discriminating these clades. With these data, we analyzed their positioning and interactions in the ASR three-dimensional models. For this purpose, we used PyMOL software and the previously modeled conformation of ASR5 from Oryza sativa (Barros et al., 2023). The same software was used to perform the alignment between three-dimensional models by the command “align” in a “one to many” or “one-to-one” mode, with five cycles of iterations and 2 Å as a cutoff. Besides, we used the modeling of free ASR5, which does not contain any ligand, and its DNA interaction form, which also incorporates zinc ions. Additionally, we used the AlphaFold Protein Structure Database (Jumper et al., 2021; Varadi et al., 2021) (https://alphafold.ebi.ac.uk/) to retrieve protein structures and models representing examples of each of the other five ASR clades. Applying the structure similarity cluster within the database, we chose the model with the highest average pLDDT (Supplementary Table S2). The chosen model was then verified to ensure it belongs to the appropriate ASR clade according to our phylogeny. Although the work of Barros et al. (2023) showed that AlphaFold modeling had lower scores due to the high number of intrinsically disordered regions present in the ASR, our preliminary analyses, regarding the ABA/WDS domain and mainly in the DNA binding portion, proved that the modeling from AlphaFold was similar to Barros’ work. Therefore, we chose to keep them, since our analysis was focused on the DNA binding portion.

Ancestral sequence reconstruction was performed using GRASP software (Foley et al., 2022), employing the LG substitution model along with the alignment and phylogeny from the Streptophyta dataset. Because accurate alignment is critical for this analysis, reconstruction was restricted to the ABA/WDS domain. Additionally, we used the estimated ancestral sequences of Charophyta to model their three-dimensional conformation. For this purpose, AlphaFold2 (via Google Colab) was utilized with three recycling iterations and minimization applied to all five models (Mirdita et al., 2022). The final model was selected as the top-ranked structure based on the pLDDT value post minimization.

## 3 Results

### 3.1 Genome-wide identification of ASR genes

**FIGURE 1:**
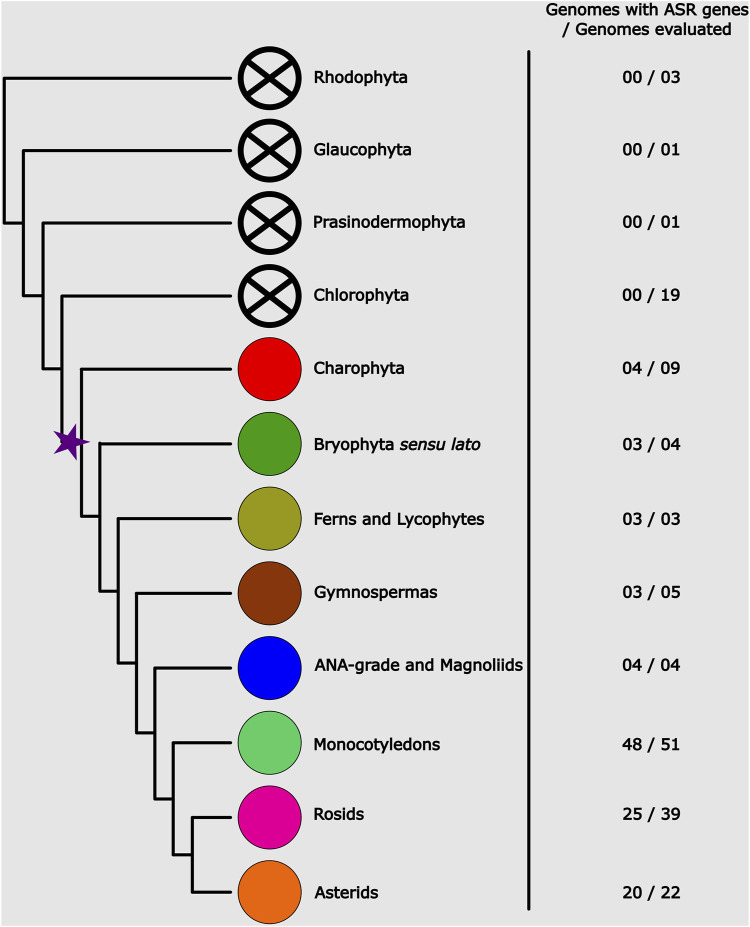
Phylogenetic representation of the Archaeplastida, showing the number of genomes sampled per group and the average number of genes present in them. The star marks the evolutionary point where the possible origin of ASR genes occurred.

From the 163 genomes analyzed, putative *ASR* genes were identified in 115. In the remaining 47 genomes, HMMER was unable to recover any *ASR* genes. The *ASR* gene possibly originated in the common ancestor of Streptophytes (which comprises Charophytes and Embryophytes) (Figure 1), since *ASR* genes have only been identified in these two lineages. No other lineage of Archaeplastida evaluated (Rhodophyta, Chlorophyta, Glaucophyta, and Prasinodermophyta) seems to encode *ASR* genes in their genomes. Notably, within Charophyta, *ASR* genes are not universally present. They are found in the genera *Klebsormidium*, *Spirogloea*, *Mesotaenium*, and *Zygnema*, but are absent in *Chara*, *Chlorokybus*, and *Mesostigma*. *ASR* genes are also not found in 16 angiosperm genomes. Among these, we highlight the Brassicaceae family, which includes the model plant *Arabidopsis thaliana*. We also found no *ASR* genes in five other Brassicaceae species. The loss of the *ASR* gene possibly occurred in the common ancestor of the Brassicaceae family, given that *ASR* genes have been found in other families of the Brassicales order. Even so, the small number of genes found in these species is also noteworthy. A single *ASR* gene was recovered in *Carica papaya* (Caricaceae) and one in *Cleome violacea* (Cleomaceae). This result suggests a reduction in the number of *ASR* genes in the Brassicales lineage, which may have facilitated the loss of the *ASR* gene in Brassicaceae.

As for the diversification of the ASR gene family, there seems to be no correlation between the number of genes and plant diversification (Figure 1; Supplementary Table S1). The high frequency of tandem duplications is noteworthy, possibly serving as the main driver of ASR gene diversification. Among all the species analyzed in this study, the three with the highest number of ASR genes were the monocotyledon *Thinopyrum intermedium* (Poaceae) with 48 putative ASR genes, followed by the bryophyte *Sphagnum magellanicum* (Sphagnaceae) and the lycophyte *Diphasiastrum complanatum* (Lycopodiaceae), both with 35 ASR genes. Among the eudicots, the species with the highest number of ASR genes identified was *Corymbia citriodora* (Myrtaceae) with 27 genes, followed by *Chenopodium quinoa* (Amaranthaceae) with 15 genes and *Eucalyptus grandis* (Myrtaceae) with 13. This result indicates a higher rate of duplications in these species, especially in Myrtaceae. On average, we found 4.17 ASR genes in the evaluated eudicot genomes. Furthermore, from the 47 eudicot species in which we identified *ASR* genes, 10 of them possibly encode for two ASR genes, and 15 only for one.

Preliminary searches were performed using BLASTp, in the Phytozome database with default options, with the OsASR5 (LOC_Os11g06720) query on the genomes of Oryza sativa v7.0 and *Solanum lycopersicum* (ITAG5.0). However, the observed e-values (∼1e-3) were relatively high, and BLASTp failed to recover all six ASR from the *O. sativa* genome. Because of this, we opted to use HMMER for the comprehensive identification of ASR across the 163 genomes studied. Notably, the BLASTp results revealed an interesting observation: the homologous region identified among the ASR proteins was practically identical across sequences (Supplementary Figure S1). Accordingly, we refer to this region as the “Core ASR”, as it exhibits the highest identity among the different ASR proteins.

### 3.2 Evolution and diversification of the ASR gene family

**FIGURE 2:**
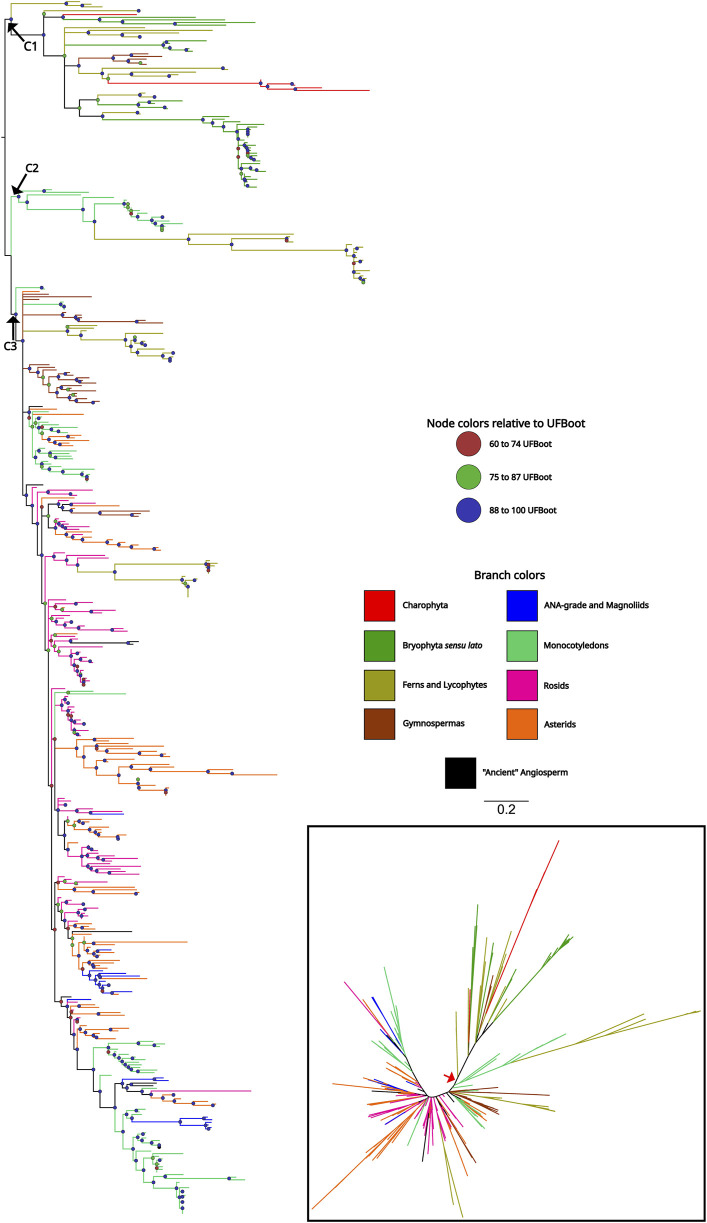
Phylogenetic tree of ASR proteins from Streptophyta. The topology was recovered after applying the maximum likelihood method, in IQTree, on the amino acid sequences of the ABA/WDS domain (Pfam ID: PF02496) that characterize ASR proteins. The colored circles on the nodes represent the Ultrafast Bootstrap obtained from 10,000 replicates. Branches with Ultrafast Bootstrap less than or equal to 59 were deleted and represented as polytomies. The phylogeny included 76 species representing the main taxa of the Streptophyta clade. Branches colors indicate which clade the sequence belongs to. The unrooted tree is represented in the lower right corner and the red arrow indicates the point where the tree was rooted.

To understand the origin and evolutionary pattern of *ASR* genes in plants, we estimated a phylogeny using a dataset including representative species from all the major groups of Streptophyta. This dataset comprises 465 sequences from 76 species (Supplementary Table S1). The rapid diversification and duplication characteristics of *ASR* genes are evident in the phylogeny, which did not converge with the evolutionary history of the species (Figure 2). The complete phylogeny can be found in the Supplementary Figure S2. We used the Charophyta sequences to root the tree. However, since these sequences did not group together, the root was placed at the node that encompasses all Charophyta sequences, allowing us to establish three major clades: The first clade (C1) is an early-diverging lineage due to the presence of ASR sequences from charophytes. In addition to charophytes, sequences from bryophytes, ferns, and gymnosperms are also grouped in this clade. Notably, there are no angiosperm sequences in this clade. In addition, it is worth noting that the sequences belonging to bryophyte species are exclusive to the C1 clade, while the ASR sequences of gymnosperms and ferns also appear in more derived clades. The second clade (C2) is the smallest in terms of both sequence number and species diversity, being the closest to the C1 clade, and it consists solely of monocots and the fern species *Ceratopteris richardii*. The C2 group also comprises the ASR6 and ASR2 genes from *Oryza sativa*. Finally, the third clade (C3) is the most diverse in terms of species and number of sequences. Eudicots and species representing the earliest lineages to diversify from angiosperms (ANA-grade and Magnoliids) are found exclusively in C3. The most striking feature of this clade is the high degree of polytomies, making it challenging to divide into subgroups (Figure 2). The PhyML results revealed a highly similar topology; however, it identified fewer well-supported groups within C3 compared to IQTree (Supplementary Figure S3).

### 3.3 Phylogeny of ASR genes shows better resolution for recent evolutionary histories

**FIGURE 3:**
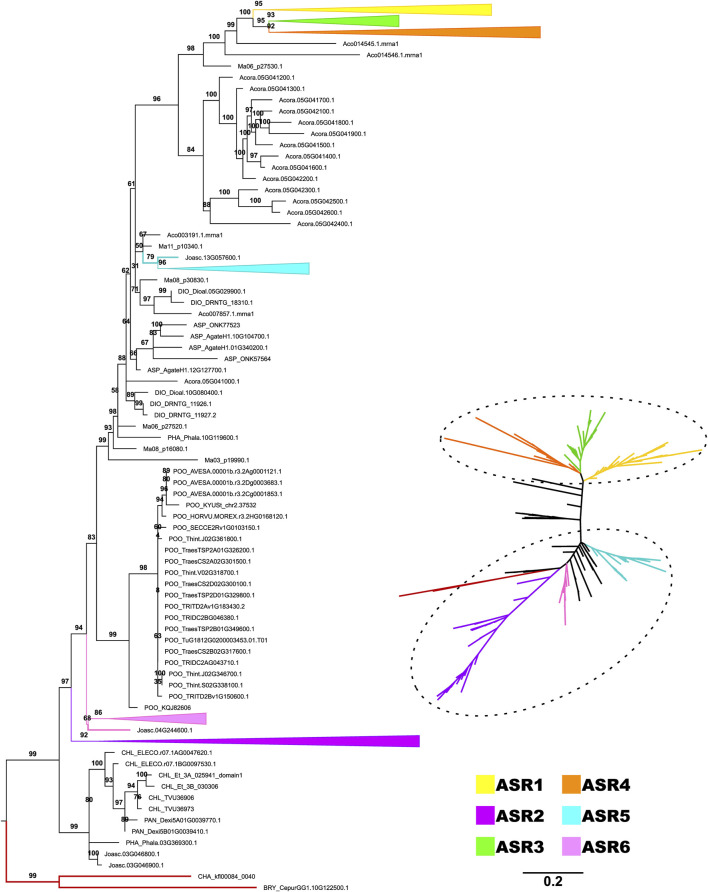
Diversification of ASR protein family from monocots. The topology was recovered after applying the maximum likelihood method, in IQTree, on the amino acid sequences of the ABA/WDS domain (Pfam ID: PF02496) that characterize ASR proteins. The phylogeny included 46 species belonging to the monocotyledon clade. The numbers on each branch represent the Ultrafast Bootstrap obtained from 10,000 replicates. The Charophyta and Bryophyta species established the root (red branches). The colors highlight the groups composed of putative proteins ASR1 (yellow), ASR2 (purple), ASR3 (green), ASR4 (gray), ASR5 (blue), and ASR6 (pastel pink), as seen in the trees rooted (left) and unrooted (right). The six ASR clades are collapsed in the rooted phylogeny.

Based on the previous result, we observed that monocot sequences are the first group of angiosperms to appear close to the early-diverging clade and, at the same time, to have sequences that are more distant from the root (Figure 2). This indication of greater differentiation of ASR in monocotyledons led us to carry out a second phylogeny focused on this group, increasing the number of species analyzed. The phylogeny of the monocotyledon dataset provided a clear definition of the evolutionary relationship between *ASR* genes. The phylogeny can be divided into two large groups, which we named as Group A and Group B (Figure 3). Uncollapsed phylogeny can be found in the Supplementary Figure S4. Group A would be the most ancestral, as it is the closest to the root, and within it, we have the clades comprising the ASR2, ASR5, and ASR6, with the *Oryza sativa* *ASR* genes as reference (Frankel et al., 2006). The ASR2 clade is the closest to the root and includes only sequences from Joinvilleaceae and Poaceae (with representation from all five subfamilies evaluated). The same pattern of species is found in the ASR5 clade, which is the most derived within Group A. Finally, the ASR6 clade appears to have diverged later and includes only Joinvilleaceae and three of the Poaceae subfamilies (Chloridoideae, Oryzoideae, Panicoideae). Between the ASR5 and ASR6 clades, there is a clade formed exclusively by sequences from the Pooideae subfamily. These sequences are putative ASR6s, as they are closely related to the rice ASR6 clade. This positioning may indicate that ASR6 of Pooiedeae has undergone greater differentiation compared to other subfamilies of Poaceae.

Group B consists of more derived ASR genes, distant from the root and forming a monophyletic group that includes ASR1, ASR3, and ASR4 proteins. Both the ASR4 and ASR3 clades contain sequences from all five Poaceae subfamilies and their sister family, the Joinvilleaceae. The ASR1 clade also includes a sequence from *Ananas comosus* (Bromeliaceae); however, sequences from the Pooideae subfamily are absent. Therefore, the Pooideae subfamily is absent from two of the six ASR clades highlighted here. Despite focusing on monocotyledons, the phylogeny’s best resolution is restricted to Poaceae and Joinvilleaceae. This result reinforces the rapid evolution of *ASR* genes, which makes it challenging to recover deeper evolutionary histories with these genes. The other seven monocot species evaluated, belonging to different orders, did not group with any defined clade. In some cases, they did not fall within the two highlighted groups (Group A and Group B), being positioned between them. Notably, most sequences from *Acorus americanus* (Acoraceae) (14 of 15) occupy this intermediate position. Additionally, two sequences from *A. comosus* (out of seven) and one from *Musa acuminata* (out of six) are also in this position. All other sequences are found exclusively within the large group of ancestral ASR, except for one single sequence from *A. comosus* grouped within the ASR1 clade.

It is worth mentioning that Group B found in monocots (ASR1, ASR3, and ASR4) remains the most distant and derived even in the phylogeny using the Streptophyta dataset. These findings suggest further differentiation and, potentially, the acquisition of new functions within this ASR group. This result reinforces the probable diversification and importance of this gene family in monocotyledons, especially in Poaceae, compared to other angiosperms. For the monocotyledon dataset, PhyML estimated a phylogeny highly similar to that obtained with IQTree, allowing the identification of the six ASR clades and their classification into Groups A and B, along with some intermediate sequences (Supplementary Figure S5).

### 3.4 Composition of motifs follows the ancestry recovered by phylogeny

Motif analysis of the Monocotyledon dataset revealed specific patterns associated with different ASR clades (Supplementary Figure S6). Motifs 0, 1, and 2 correspond to the ABA/WDS domain, which is conserved across all clades. Motif 6 is exclusive to group A (early-divergent clade), although it is not universally present. Similarly, Motif 10 is exclusive to group A, except for three sequences from group B. It is located in the C-terminal portion and it is absent in the ASR2 clade. In contrast, Motif 8, while also present in the C-terminal portion, is exclusive to group B (derived clade), with only three sequences from group A exhibiting this motif. Motif 3 corresponds to the putative zinc-binding domain and shows a scattered distribution, albeit with enrichment in the ASR5 and ASR1 clades. Motif 11 occurs multiple times within the same protein and is enriched in the ASR6 clade, being shared between the ASR2 and ASR6 clades. Motifs 9, 7, 4, and 13 are restricted to the Pooideae family. Motif 9 is enriched in ASR3 genes and in some members of the ASR2 clade, whereas the remaining motifs are generally exclusive to the ASR2 clade, with few exceptions. Motif 12 is also enriched in Pooideae, being found in the ASR3 and ASR2 clades, though not universally. The last two motifs are clade-specific, with fewer than three exceptions: Motif 14 is exclusive to the ASR6 clade, while Motif 5 is exclusive to the ASR2 clade.

Considering the Streptophyta dataset, the C1 early-divergent lineage encompasses sequences with aggregated motives compared to the other two lineages, and an overall Motif 11-5-4-1-0-9 organization (Supplementary Figure S7). It is noteworthy the replacement of Motif 3 for Motif 11 in the N-terminal portion of two ancestral sequences (BRY_Sphmag05G052700.1 and BRY_Sphmag01G112900.1) to reach the above mentioned pattern, and the acquisition of Motif 4. Besides, the sequences without Motifs 2 or 9 in the C-terminal portion are made of Motifs 6 or 7 in exchange. On the other hand, the C3 clade encompasses less-structural diverse sequences composed mainly of core Motifs (ABA/WDS) and Motifs 3 and 6. Other members mark the expansion of Motif 10 and a progressive introduction and expansion of Motif 13 in the C3 clade, where one of four sequences bear two copies of Motif 13. The change is noticed in the C2 clade, whose sequences have up to three copies of Motif 13 and are the only ones with Motifs 12 and 14, besides the predominance of Motif 8 (except by three sequences in C3).

### 3.5 ASR clades are formed by proteins with active sites in the DNA-binding domain

**FIGURE 4:**
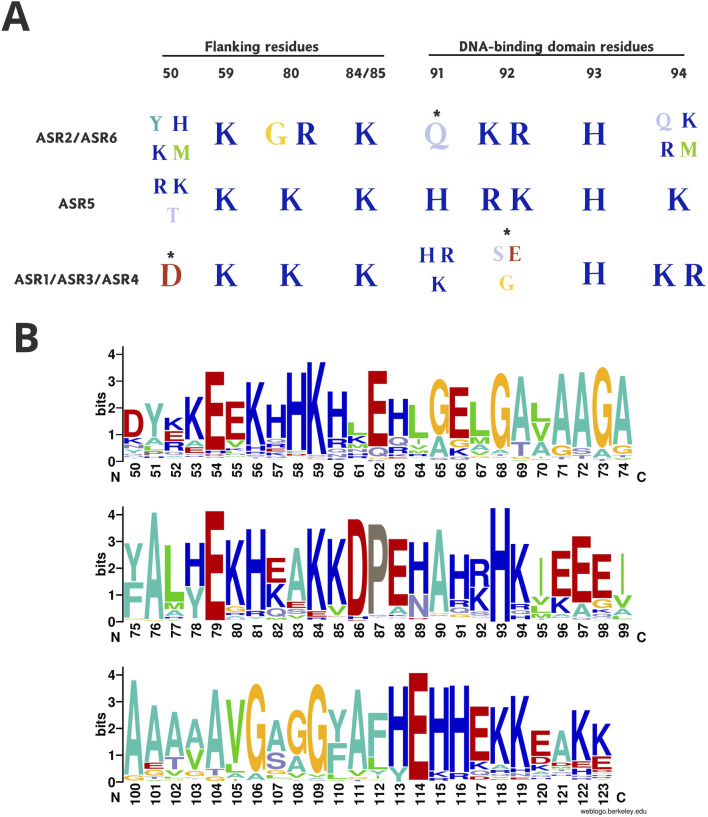
Comparative residue composition between ASR proteins. The illustration in **(A)** depicts the dynamics of residue substitutions between ASR groups of monocots according to the diversification of each, that is, from the most ancestral (ASR2, ASR6, and ASR5) to the most derived (ASR1, ASR3, and ASR4) ones. The highlighted positions belong to flanking sites (50, 59, 80, 84, and 85) and the DNA-binding domain (positions 91–94) predicted by Barros et al. (2023) as crucial for the OsASR5 transcription factor activity. Asterisks (*) mark positions not occupied exclusively by the residues presented. The sequence logos **(B)** depicts the composition of the ABA/WDS (Pfam ID: PF02496) of all ASR proteins belonging to the Streptophyta dataset. In the plot, the x-axis points out the positions in the sequences, and the y-axis represents the amplitude of each residue, which is proportional to the conservation in the alignment between the sequences.

The composition of the recovered clades’ DNA-binding domain showed a predominance of Q residues at position 91 in the early diverged clades of ASR proteins, formed by ASR2 and ASR6 sequences (Figure 4A). On the other hand, the intermediate ASR5 clade marks the transition to the emergence of positively charged residues at the same position, as noticed among the more recent ASR1, ASR3, and ASR4 clades. Moreover, the divergent clades also highlight the transition in the pattern of positive residues found in the first-divergent proteins (ASR2 and ASR6) at position 92, to a more diverse class of amino acids, represented by residues such as S, G, and E, in the other clades. In contrast, the amino acid at position 93 is exclusively a H across all recovered groups. Finally, a tendency towards positive residues was observed in most clades, where K and R residues are found at position 94, except by the ASR2 clade, where Q and M residues are conserved.

Regarding the residues flanking the DNA-binding domain, which are also crucial for the stability of the complex with the target to be regulated (Barros et al., 2023), the sequence logos highlight prominent patterns: the derived clades ASR1, ASR3, and ASR4 have acquired a D at position 50, while K is conserved at positions 59 and 85 across all clades. In the first diverged clade, ASR2, residue position 89 is mainly occupied by G, a non-polar residue. This glycine is replaced by R, a positively charged amino acid residue, in ASR6. This class of amino acids became predominant in the other clades, which are exclusively formed by K in this position.

As expected, the residues found in the ABA/WDS domain within the Streptophyta dataset are more diverse than those in the Monocotyledon dataset. Besides H^93^, found in the DNA-binding domain, only two E residues, at positions 79 and 114, and an A, at position 76, are conserved in most of the evaluated sequences (Figure 4B). After setting the ASR5 from *Panicum hallii* as a reference, the alignment between this three-dimensional model and other representatives of the remaining five ASR clades of Monocots, modeled by AlphaFold, showed RMSD values of 0.23 Å (ASR1, 288 atoms), 0.38 Å (ASR3, 342 atoms), 0.41 Å (ASR4, 338 atoms), and 0.41 Å (ASR6, 316 atoms). These results corroborate the conservation of the ABA/WDS domain among these paralogs despite the disparity of the OsASR2 model (RMSD 8.76 Å, 433 atoms) (Supplementary Figure S8). Consider checking out Supplementary Table S2 for clarification about the above-mentioned representatives of each ASR clade. Additionally, for a better understanding of ASR protein domain organization, consider looking for the distribution and residue composition of each domain in light of a rice ASR5 protein as a reference in Supplementary Figure S9. Interestingly, even the reconstructed ancestral sequence of the ABA/WDS domain (KAKKEEKKHKRNELMAGVGALAAGGFAAWEAHEAFVDPGHAKKHKMEAGVAGAVAVGAGGYALHEHHEKKKLEK) exhibited a fold highly similar to those observed in the six ASR clades mentioned above. The RMSD values ranged from 0.35 to 0.79 Å, with the exception of ASR2, which showed a significantly higher value of 8.6 Å. The reconstructed ancestral domain had an average pLDDT value of 85.

### 3.6 The ABA/WDS domain is under purifying selection in Poaceae ASR genes

Our analyzes using our dataset did not reveal any sites under positive selection in the Poaceae ASR genes. The phylogeny used to perform the site model test is found in Supplementary Figure S10. The estimated ω values based on the M8 model indicates a predominance of purifying selection, with values ranging from 0.050 to 0.357 (Supplementary Figure S11). We observed that the highest ω values occurred at the extremities of the ABA/WDS domain (both C and N terminal). These peaks suggest that these regions experience lower selective pressure relative to other sites, thereby permitting greater residue variability, albeit still limited (indicated by the low ω values). Particularly interesting are the peaks in the central region of the domain, specifically at sites 91 and 92, which are located within the DNA binding site. These residues may contribute to binding specificity, facilitating the recognition of different DNA motifs.

## 4 Discussion

Evolutionary studies of *ASR* genes are limited, with the first and most comprehensive one conducted by Frankel et al. (2006). Our work applies broad sampling and robust phylogenetic methods, such as maximum likelihood, to investigate the evolution of *ASR* genes. Prior to the review by González and Iusem (2014), ASR genes were only described in Embryophytes, a clade that includes Gymnosperms and Angiosperms. Our study identifies *ASR* genes in early groups of land plants, such as mosses and ferns.

The likely origin of the *ASR* genes lies in the common ancestor of Charophytes and Embryophytes, as we did not detect their presence in Chlorophyta genomes (Figure 1; Supplementary Table S1). Since *ASR* genes are involved in response to desiccation and are ABA-responsive (Yang et al., 2005; Sachdeva et al., 2020), they might have played a role in the adaptation of plants to terrestrial environments (Delwiche and Cooper, 2015; Kapoor et al., 2022). One of the points highlighted by Frankel et al. (2006) was the greater similarity among ASR paralogues when compared to their orthologues. These observations are in line with the hypothesis of the functional redundancy between OsASR1 and OsASR5 proposed by Arenhart et al. (2012), which are co-localized in the nucleus and cytoplasm, and whose conformational pattern is highly similar (Supplementary Figure S12). Our results corroborate this observation, as the phylogeny showed low support at deep nodes (Figure 2). The C3 clade is a clear example, exhibiting numerous polytomies and well-supported minor subclades. These smaller subclades are generally composed exclusively of sequences derived from species within the same botanical family and, in certain cases, from the same order.

The distribution of genes suggests that the process of tandem duplications is the main responsible for the expansion of the ASR family. From the 465 sequences analyzed in the Streptophyta dataset, 340 came from tandem duplications [they are found on the same contig/chromosome separated by less than 100 Kb (Liu et al., 2021)]. Since 21 are unique sequences (one ASR in the genome), the other 104 came from other duplication mechanisms.

Frankel et al. (2006) pointed out that ASR2 and ASR6 from *Oryza sativa* were the most divergent compared to other ASR genes analyzed. This statement is corroborated and reinforced by our analyses. In the Streptophyta dataset, the C2 clade is the closest to the ancestral ASR genes (Figure 2), consisting mostly of monocot sequences, which include ASR2 and ASR6 from rice (Supplementary Figure S2). The other four ASR sequences from rice are grouped in C3. For ASR6, the *O. sativa* ssp. japonica sequence (LOC_Os04g34600) is the only one in the clade, within the Oryzoideae subfamily, that contains the ABA/WDS domain incomplete. Even though the *O. sativa* ssp. indica genome contains ASR6 (BGIOSGA015105) with the complete domain. This finding indicates a fast accumulation of mutations in ASR6 genes during the differentiation of the *O. sativa* ssp. japonica (Londo et al., 2006; Campbell et al., 2020). However, the possibility that this could be a sequencing/assembly error can not be ruled out, due to the presence of seven uncertain nucleotides (N) in the first exon (O’Rawe et al., 2015). For tomato ASR, the five genes were recovered, and as expected, four of them are grouped in the same subclade in C3, along with other sequences from Solanaceae (Supplementary Figure S2).

The pLDDT values serve as a measure of confidence in the models generated by AlphaFold2. This scale ranges from 0 to 100, where values exceeding 90 indicate high precision, values between 70 and 90 suggest good precision, and values below 70 denote lower confidence, with those below 50 often indicating intrinsically disordered regions (Middendorf and Eicholt, 2024). The overall average pLDDT of representatives from each selected clade shows that only ASR1 has a confident prediction, reflecting the intrinsically disordered nature of the ASR proteins. Nevertheless, our study focuses on the “ABA/WDS induced protein” domain. When considering the pLDDT values solely for the domains, only ASR2 and ASR6 exhibit values below 70; furthermore, when evaluating just the core of the domain, only ASR2 demonstrates low confidence (Supplementary Table S2).

Some ASR proteins have already been characterized as intrinsically disordered, including the “ABA/WDS induced protein” domain. Experiments with two representatives of the ASR5 clade revealed that they undergo conditional folding, meaning that under certain conditions, they adopt tertiary structures (Hamdi et al., 2017). The authors tested glycerol (which mimics dehydration), presence of zinc ions, and 2,2,2-trifluoroethanol (TFE), which mimics the effects of a protein’s hydrophobic interactions with its target (Hamdi et al., 2017). Conditional folding was similarly reported for tomato ASR1 under dehydration conditions and in presence of zinc ions (Goldgur et al., 2006). Furthermore, a previous *in silico* study by our group demonstrated that the addition of zinc ions stabilizes ASR and promotes the formation of secondary structures (Barros et al., 2023). Moreover, we observed a strong similarity between this model and those predicted by AlphaFold, particularly in the core of the domain. Proteins are dynamic, constantly changing conformations, and the AlphaFold prediction represents a single snapshot of the protein’s conformational landscape (Guo et al., 2022). A study shows that AlphaFold2 can systematically identify disordered regions that present conditional folding (Alderson et al., 2023). Based on these results, we propose that the core of the domain adopts a functional helix–turn–helix fold, a structural motif well documented in the literature and associated with DNA binding.

**FIGURE 5:**
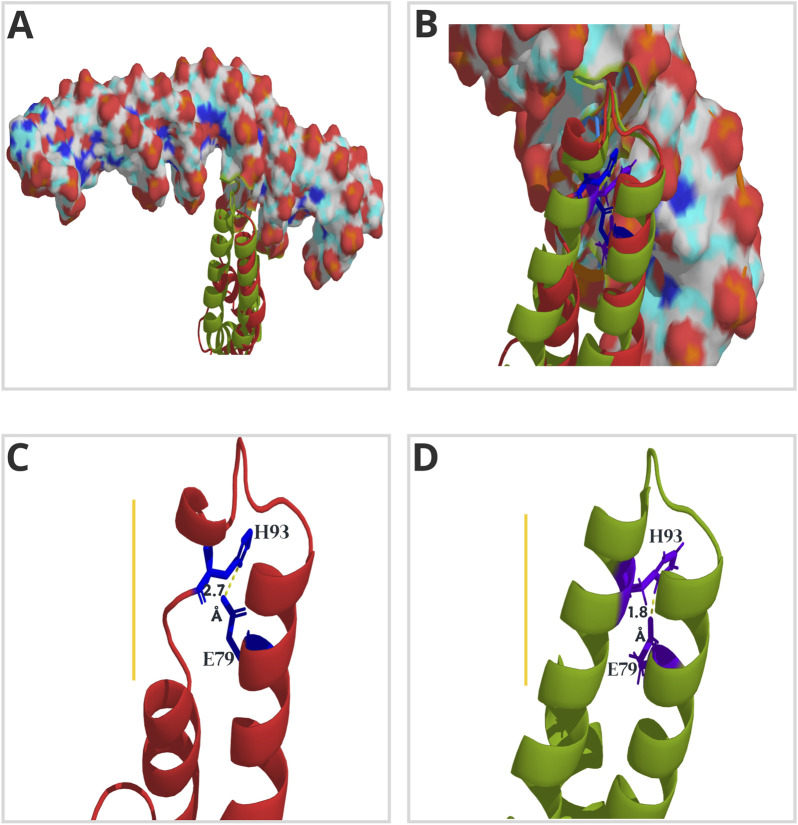
Three-dimensional (3D) model of ASR5 protein from rice (*Oryza sativa* ssp. *japonica*). The lateral view highlights the α-helices and loops that form the DNA-binding domain, as proposed by Barros et al. (2023). The model in red shows the free OsASR5 protein, while the one in green represents the protein bound to the *cis*-element (illustrated in surface mode) of the promoter region of the *STAR1* (Barros et al., 2023). Both conformations are aligned in **(A)** and **(B)** to emphasize the structural gain during OsASR5-*STAR1* complex formation. In **(C)** and **(D)**, the distances in Angstrom (Å) between residues H93 and E79 (illustrated in stick mode) in the free (red) and bound (green) versions of OsASR5, respectively, are illustrated. The yellow line points out the relevant structural differences.

Despite the reasonable level of amino acid conservation in ASR sequences, only four residues are highly conserved (A^76^, E^79^, H^93^, and E^114^) throughout all clades (Figure 4B). Glutamic acid (E) commonly increases the accessibility of proteins to the solvent (Uversky, 2013). This parameter contributes to the determination of binding hotspots (Barik et al., 2015), as it is associated with the formation of complexes (Chakravarty et al., 2013). The H^93^ residue is critical for OsASR5 binding to the *STAR1* target (Barros et al., 2023). From the alignment between the free and bound OsASR5 models (Figures 5A,B), we observed that the α-helix involved in DNA binding, and containing the H^93^, is missing in the free OsASR5 conformation (Figure 5C, yellow line) compared to the bound one (Figure 5D). We also noticed that E^79^ interacts with H^93^ and are closer to each other (1.8 Å) in the bound OsASR5 model (Figure 5D). Therefore, E^79^ residue can potentially contribute to ASR stability when binding to DNA, allowing the α-helix establishment. Nevertheless, site-directed mutagenesis experiments should be performed to confirm such hypotheses.

In terms of the monocots ASR groups, the main amino acids involved in DNA-binding display different sequence consensus (Figure 4A; Supplementary Figure S13). The first-divergent ASR proteins in Group A (ASR2 and ASR6) are mostly composed of E at position 91, and basic amino acids at position 92. Conversely, Group B contains basic amino acids at position 91, and a greater diversity at 92, containing polar and non-polar residues (Figure 4A). This variety in the DNA-binding domain can imply an increased diversification in which *cis*-elements the ASR proteins can recognize and regulate, as the different residues interact distinctly with DNA bases (Hossain et al., 2023). Functional studies using the ChIP-seq approach would pave the way to understanding how ASR proteins differentiate themselves, regarding the promoters they bind and which set of genes they regulate (Ricardi et al., 2014).

Regarding the composition of the sequences, there is a conservation of residues that promote structural disorder, such as E, H, K, A, and G (Romero et al., 2000), and a low frequency of hydrophobic residues among ASR proteins of the Streptophyta dataset (Figure 4B). In addition to this evidence, the structural disorder is also revealed by the difficulties in building multiple alignments (Riley et al., 2023), such as what occurred during the assembly of our Streptophyta dataset. It is important to highlight that the predominance of charged residues, as observed among ASR proteins, favors the activity of molecular chaperones (Volkin et al., 1993; Narberhaus, 2002), and the enrichment of E promotes interaction with histones (Uversky, 2013), as noticed in grape ASR (Atassanov et al., 2022).

We hypothesized that ASR proteins are versatile and that the ancestral ones worked as molecular chaperones. According to Rebeaud et al. (2021), the increase in quality control required by the expansion of eukaryotic proteomes did not occur through the diversification of new families of core chaperones but rather through the duplication of existing members. Moreover, the tendency towards purifying selection of mutations that destabilize protein conformations (Smock et al., 2016), the lack of investment in sequence variability for interaction with new substrates due to the reduced binding specificity of chaperones, the uniformity between types of substrates (Bogumil et al., 2013), and the compensatory effects between copies may have contributed to the neofunctionalization and diversification of regulatory ASR proteins.

Since the duplication of transcription factors can be followed by evolutionary novelties in regulatory networks (Voordeckers et al., 2015), mutations in these proteins produce adverse pleiotropic effects (Hsia and McGinnis, 2003), in contrast to those that affect only *cis*-regulatory elements (Wray, 2007). Because of this, the conservation of the ABA/WDS domain, in addition to functional evidence (Arenhart et al., 2013; Dominguez and Carrari, 2015; Arenhart et al., 2012), corroborates the role of ASR proteins as transcriptional regulators, whose family expansion is essentially based on duplications so that the original copy maintains the stability of the regulatory network, while the other is subject to greater evolutionary flexibility, resulting in subfunctionalization, such as responses to different environmental cues, or neofunctionalization (Lynch and Force, 2000; Conant and Wolfe, 2008).

Additionally, the existence of few ASR genes among unicellular eukaryotes and the noticeable abundance of them in higher plants suggest involvement with the occupation of the terrestrial environment (Kapoor et al., 2022), and it corroborates their role as TFs assigned to some members of this family (Arenhart et al., 2013; Dominguez and Carrari, 2015; Arenhart et al., 2012) since this class of proteins is more abundant in plants compared to other eukaryotes (Shiu et al., 2005).

## 5 Conclusion

*ASR* genes seem to have emerged before the rise of Charophytes and evolved dynamically in different groups, or even in a species-specific manner, which encompasses several tandem duplications and gene losses. Regarding Angiosperms, Poaceae is the only family possessing *ASR* genes that are evolutionarily closer to their ancestors, in addition to their presence in the most derived clades, indicating a greater importance of *ASR* genes in Poaceae. Regarding ASR proteins from monocots, we highlight the DNA-binding site, particularly residues 91 and 92. These residues exhibit greater variability compared to residues 93 and 94, as indicated by the estimated ω values. This suggests that sites 91 and 92 may play a role in recognizing different DNA motifs, which has yet to be tested. Gene functional analyses are still necessary to unveil which set of genes the divergent ASR can regulate, or whether they are actually functionally redundant. Furthermore, we proposed the structural importance of the E(X)_14_H motif within the ABA/WDS domain, based on the conservation of these residues and the three-dimensional model of ASR.

## Data Availability

The original contributions presented in the study are included in the article/Supplementary Material, further inquiries can be directed to the corresponding authors.

## Supplementary material

SUPPLEMANTARY TABLE S1 Species subjected to genome-wide identification of ASR genes. This table includes the taxonomic classification of each species, the prefixes used to identify the sequences in their respective datasets, the number of genes initially recovered, and the number retained after refinement and preliminary analyses. Genomes with no recovered ASR genes are highlighted in purple. Genomes that lacked ASR genes after filtering are highlighted in yellow. Monocotyledon genomes included in the Streptophyta dataset are highlighted in light green.

SUPPLEMANTARY TABLE S2 Access code for models retrieved from the AlphaFold Protein Structure Database, which ASR clade in monocotyledons it represents, species name, and average quality value of the modeled protein (pLDDT), for the domain, and for the “core portion”.

SUPPLEMANTARY TABLE S3 Sequence acronyms used in the phylogenies estimated by PhyML and their corresponding acronyms in IQTree. The modifications were made to comply with the ten-character header limit imposed by PhyML.

SUPPLEMENTARY FIGURE S1 Results of BLAST using OsASR5 as the query. Schematic representation of the regions where BLASTp returned hits in the genome of *Oryza sativa* and *Solanum lycopersicum*, along with the corresponding e-values.

SUPPLEMENTARY FIGURE S2 Phylogram of the ASR gene family in Streptophytes. Maximum-likelihood phylogeny, estimated by IQTree, containing 76 species, with representatives of the main clades of Streptophytes. The phylogeny was estimated from proteins, using the portion of the ABA/WDS induced domain and the model LG+I+R5. Branches colors indicate which taxonomic group the sequence belongs to. UltraFast Bootstrap values are indicated in their respective branches.

SUPPLEMENTARY FIGURE S3 Phylogram of the ASR gene family in Streptophyte, estimated by PhyML using the model LG+I+R5. Phylogeny was estimated from proteins, using the ABA/WDS-induced domain portion. Branch colors indicate to which taxonomic group the sequence belongs. Bootstrap values are indicated in their respective branches. Correspondence codes are found in Supplementary Table S3.

SUPPLEMENTARY FIGURE S4 Phylogram of the ASR gene family in Monocotyledons. Maximum-likelihood phylogeny of monocotyledons, estimated by IQTree, based on the peptide sequence of the ABA/WDS induced domain and using the model Q.plant+R5. UltraFast Bootstrap values are indicated in their respective branches. A sequence of Charophyta and a sequence of Bryophyta were used to root the tree, and its branches are highlighted in red. The ASR1 clade is highlighted in yellow, ASR2 in purple, ASR3 in green, ASR4 in orange, ASR5 in blue, and ASR6 in light pink.

SUPPLEMENTARY FIGURE S5 Phylogram of the ASR gene family in Monocotyledons, estimated by PhyML using the model JTT+R4. Phylogeny was estimated from proteins, using the ABA/WDS-induced domain portion. A sequence of Charophyta and a sequence of Bryophyta were used to root the tree, and its branches are highlighted in red. The ASR1 clade is highlighted in yellow, ASR2 in purple, ASR3 in green, ASR4 in orange, ASR5 in blue, and ASR6 in light pink. Correspondence codes are found in Supplementary Table S3.

SUPPLEMENTARY FIGURE S6 Protein motifs of the Monocotyledons ASR gene family. The Pooideae sequences, although not grouped into the ASR6 clade, show similarity in the composition of the motifs with it. Therefore, we consider them as putative ASR6.

SUPPLEMENTARY FIGURE S7 Protein motifs of the Streptophytes ASR gene family.

SUPPLEMENTARY FIGURE S8 Three-dimensional (3D) models of ASR proteins representatives of each Monocot ASR clade. The lateral view shows the alignment between the models of an ASR1 (cyan), ASR2 (yellow), ASR3 (purple), ASR4 (orange), ASR5 (magenta), and ASR6 (black) modeled by AlphaFold, emphasizing the conservation of the ABA/WDS domain (Pfam ID: PF02496).

SUPPLEMENTARY FIGURE S9 Domain organization and regions of interest of a rice ASR5 protein. The OsASR5 three-dimensional (3D) model in **(A)** highlights, by a color code, the putative metal-binding site (green), the ABA/WDS domain (black) scrutinized by this study, which encompasses the core peptide (light blue) conserved among ASR proteins, including the DNA-binding domain (DBD) (dark blue). The color red represents the remaining regions of OsASR5. The supporting schematic illustration depicted in **(B)** labels each domain following the color code, along with the **(C)** protein sequence of OsASR5.

SUPPLEMENTARY FIGURE S10 Phylogram representing the ASR genes sub-dataset used in CodeML. The phylogeny was estimated by IQTree using the model TIM3+F+R5 and the nucleotide sequences. The phylogram was rooted only for visualization. The unrooted tree was used for CodeML. The arrow indicates the branch where the tree was rooted.

SUPPLEMENTARY FIGURE S11 Posterior mean ω for each amino acid site within the ABA/WDS domain. These values were estimated using the M8 model in EasyCodeML, based on ASR gene sequences from Poaceae and Joinvilleaceae. Site numbering corresponds to the position of the ABA/WDS domain in ASR5 of Oryza sativa (LOC_Os11g06720).

SUPPLEMENTARY FIGURE S12 Three-dimensional (3D) models of ASR1 and ASR5 proteins from rice (*Oryza sativa* ssp. japonica). The lateral view shows the alignment between the OsASR1 (cyan) and OsASR5 (magenta) models provided by AlphaFold.

SUPPLEMENTARY FIGURE S13 Comparative residue composition between monocot ASR protein groups. The sequence logos depict the composition of the ABA/WDS (Pfam ID: PF02496) of the **(A)** ASR1, **(B)** ASR2, **(C)** ASR3, **(D)** ASR4, **(E)** ASR5, and **(F)** ASR6 clades. In the plot, the x-axis points out the positions in the sequences, and the y-axis represents the amplitude of each residue, which is proportional to the conservation in the alignment between the sequences.
