# Supplementary figures and images for: ASR gene family: a case of tandem-drive evolution
Source: Front Mol Biosci. 2025 Jun 13;12:1456645. doi: 10.3389/fmolb.2025.1456645 (PMC12202234; doi:10.3389/fmolb.2025.1456645)

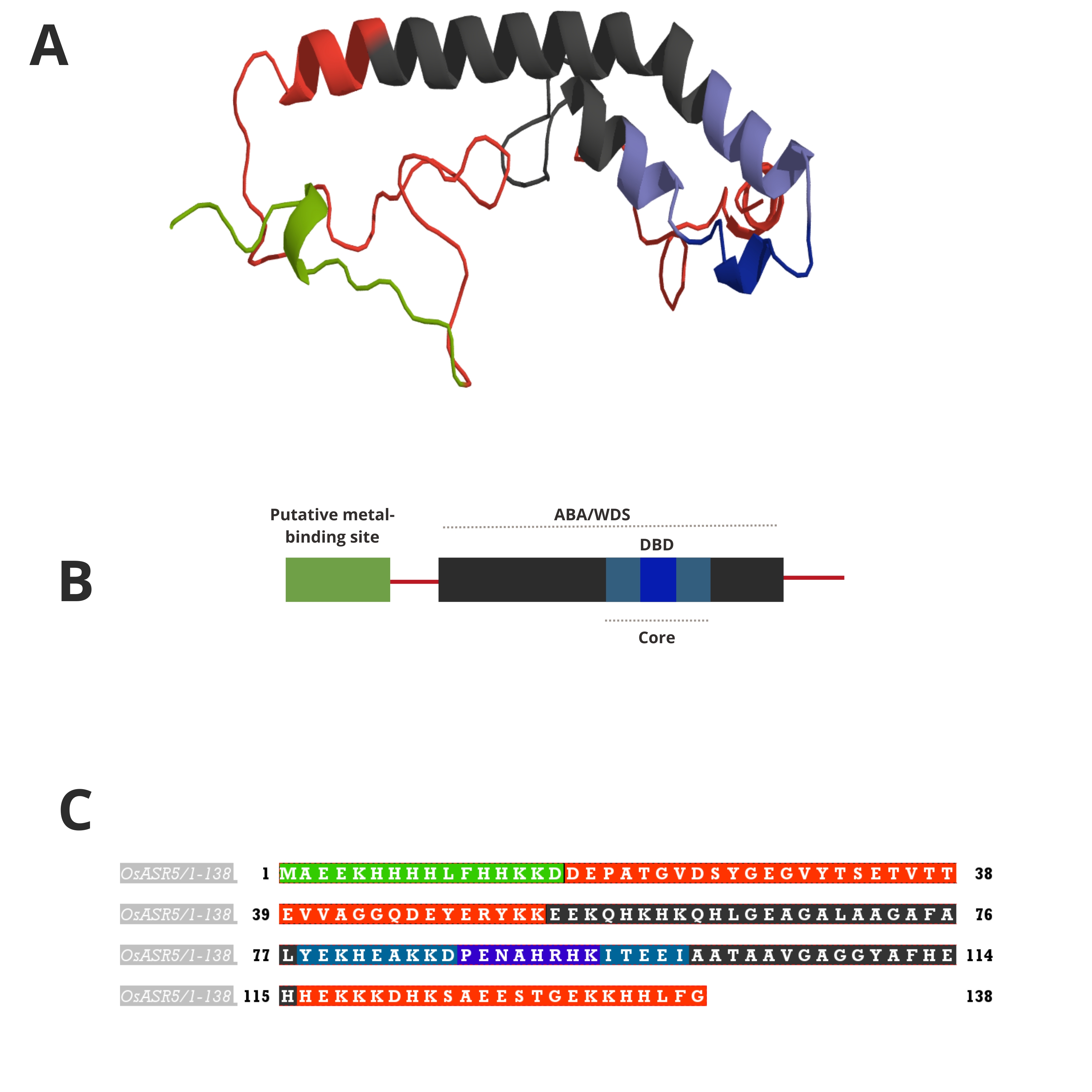

Supplement: Supplementary file 5 [file Image9.jpeg]

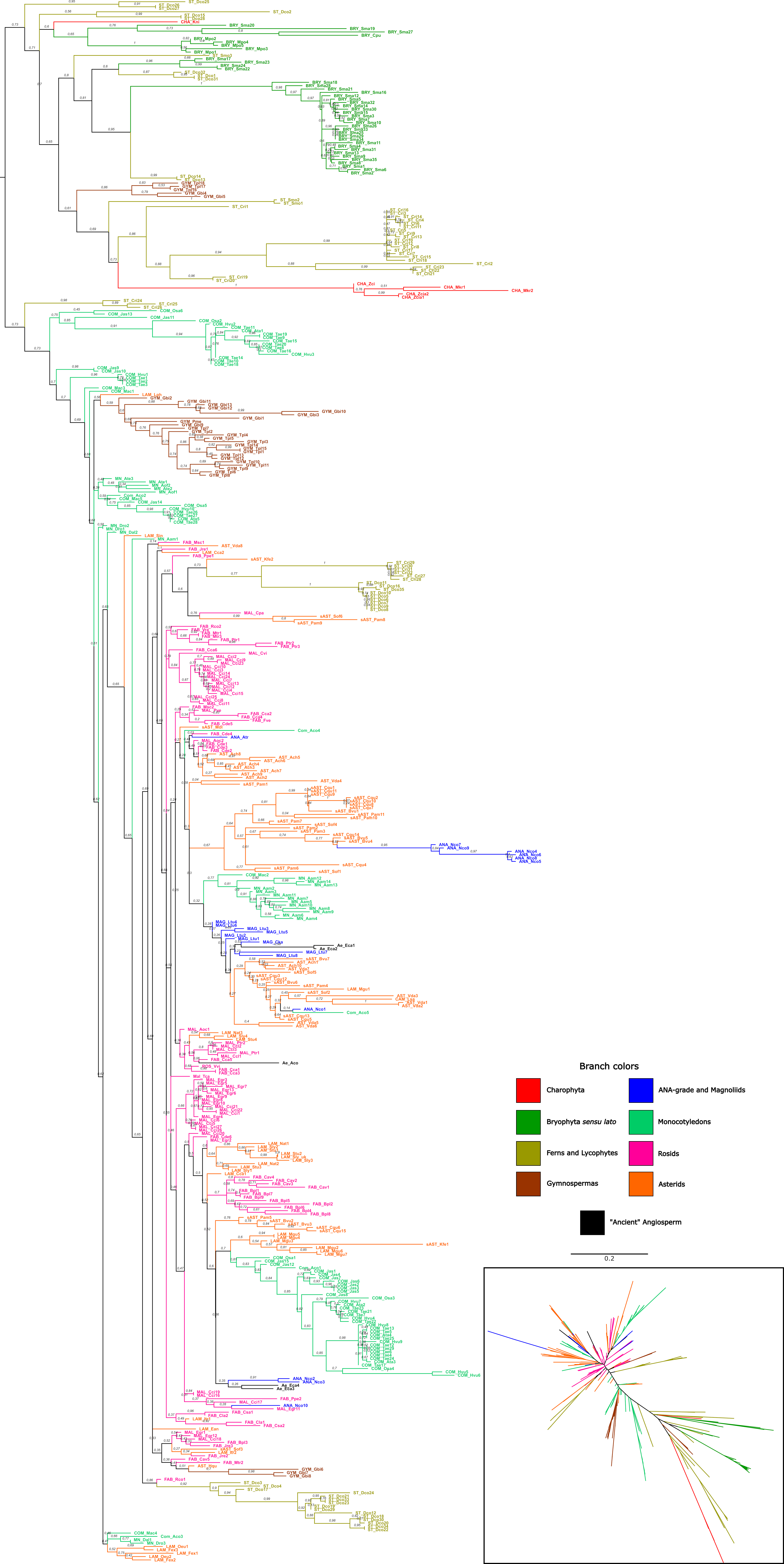

Supplement: Supplementary file 6 [file Image3.tif]

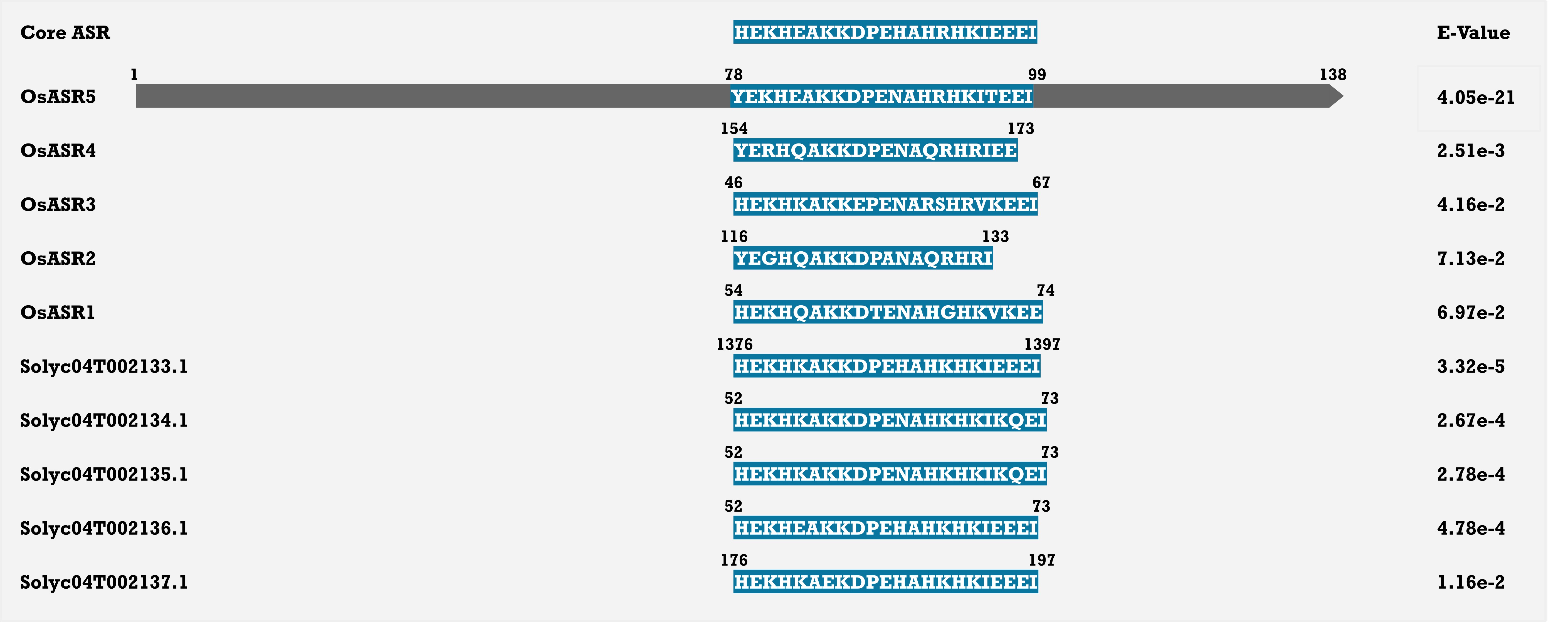

Supplement: Supplementary file 7 [file Image1.jpeg]

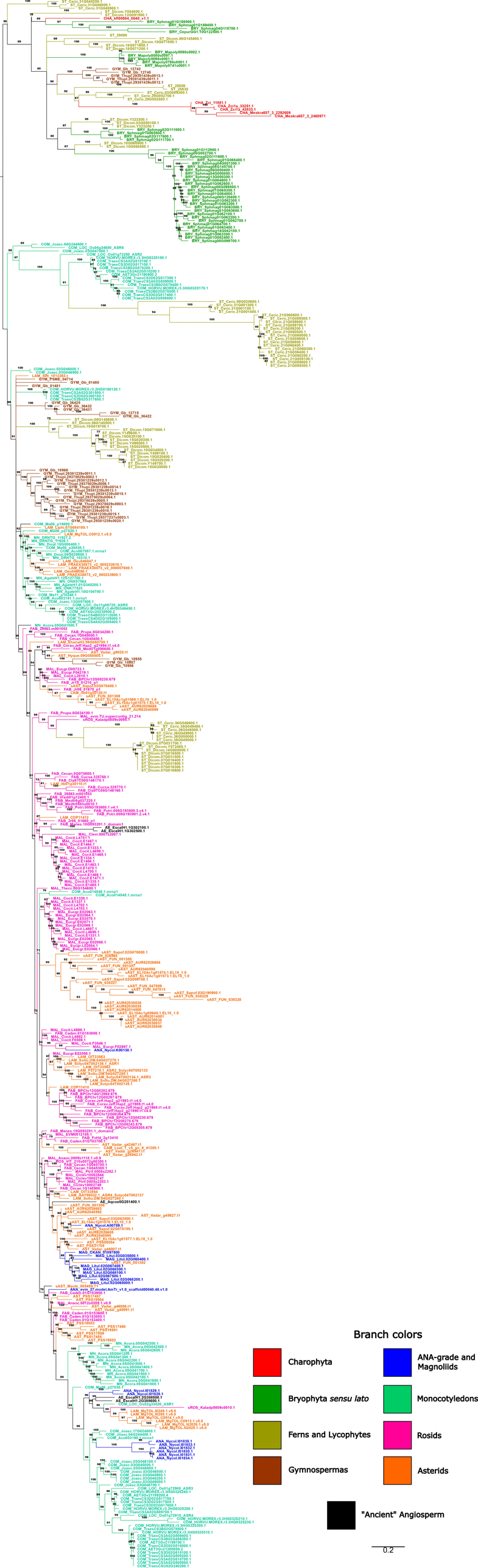

Supplement: Supplementary file 9 [file Image2.jpeg]

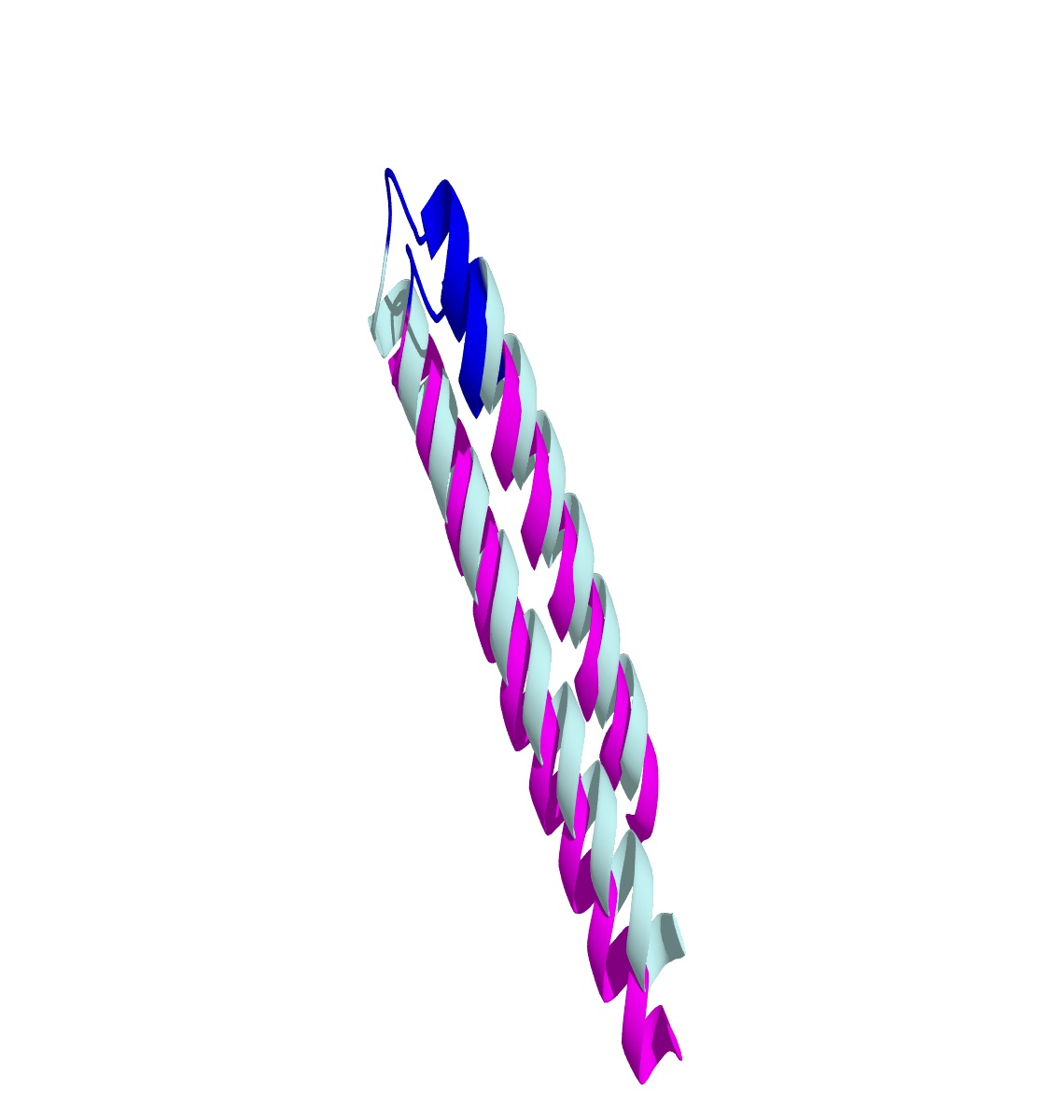

Supplement: Supplementary file 11 [file Image12.jpeg]

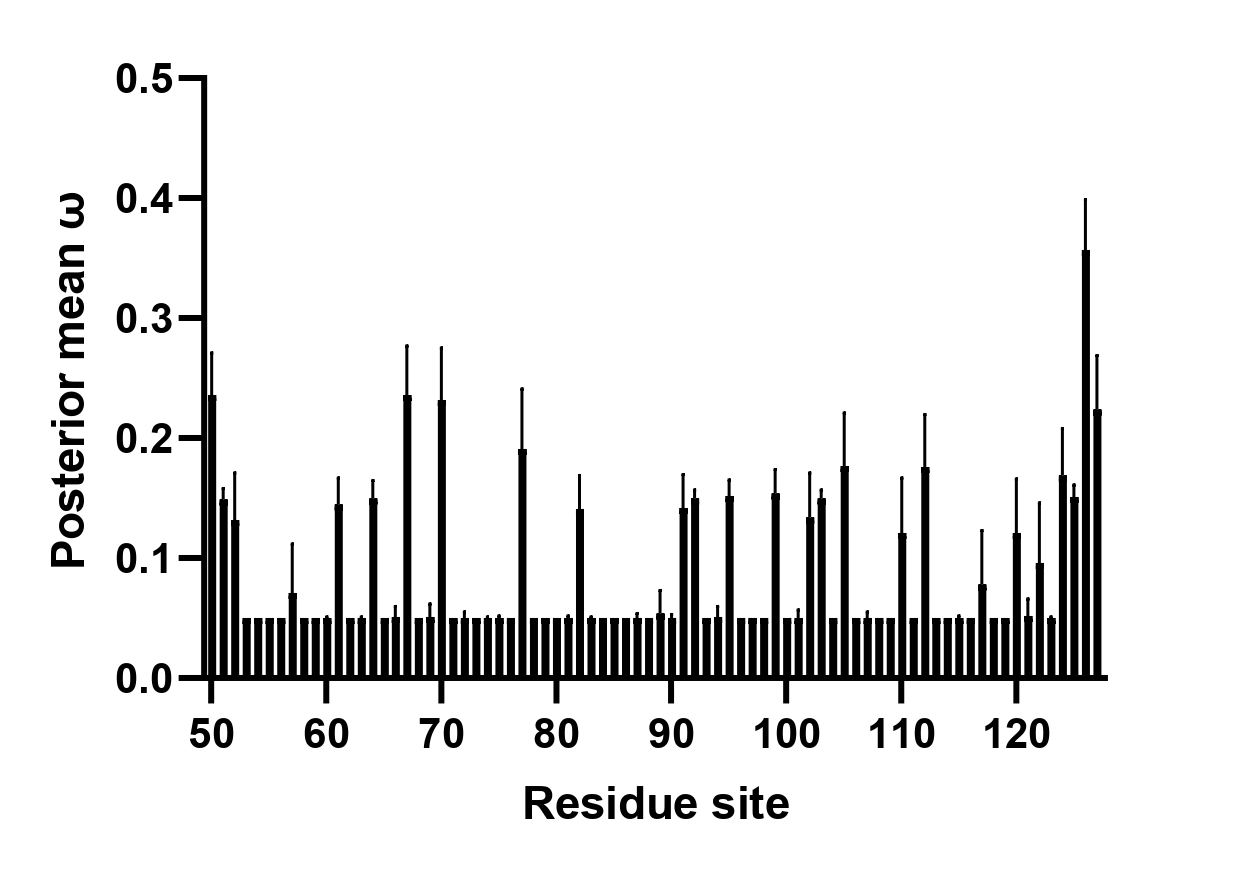

Supplement: Supplementary file 12 [file Image11.jpeg]

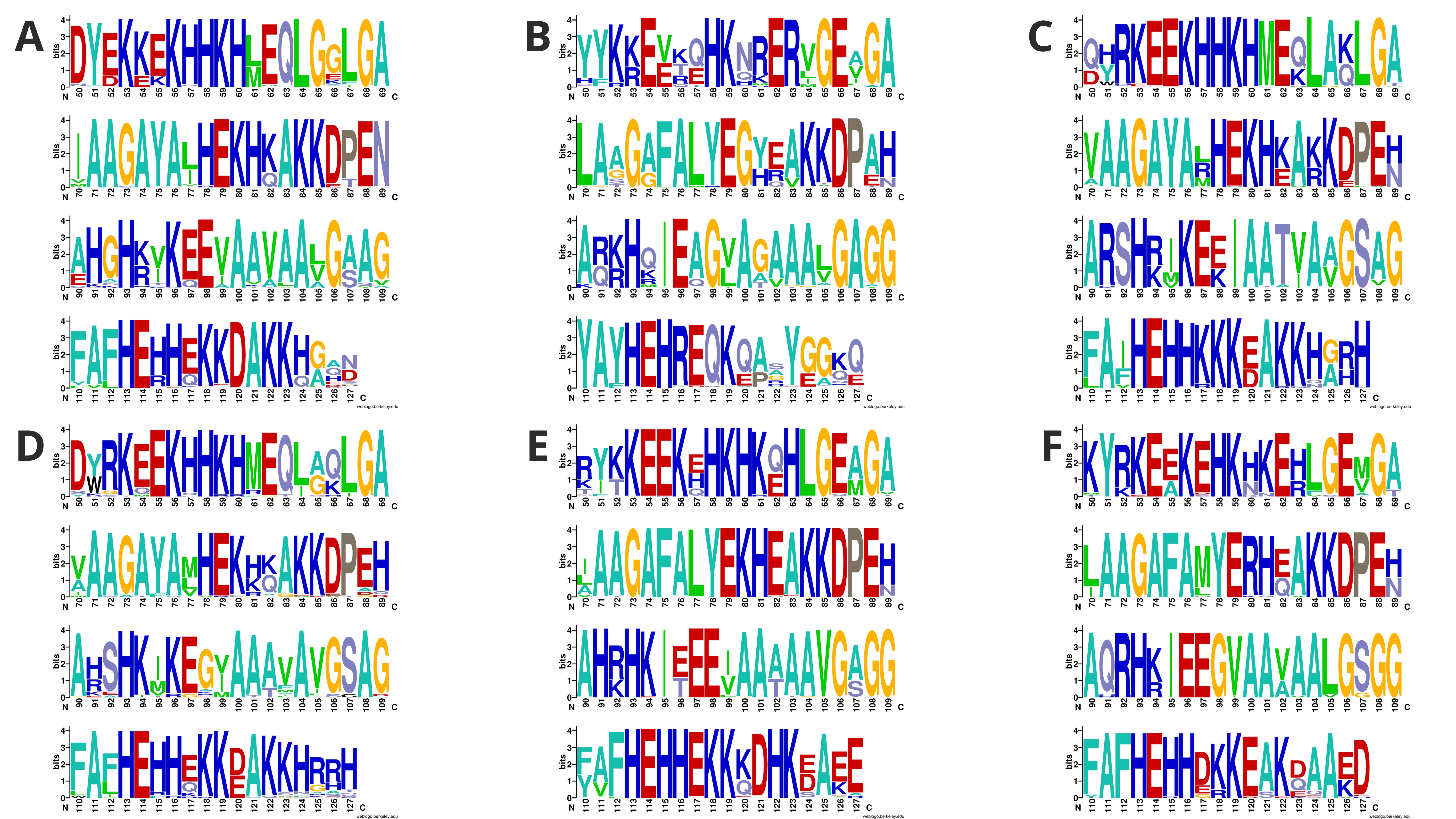

Supplement: Supplementary file 15 [file Image13.jpeg]

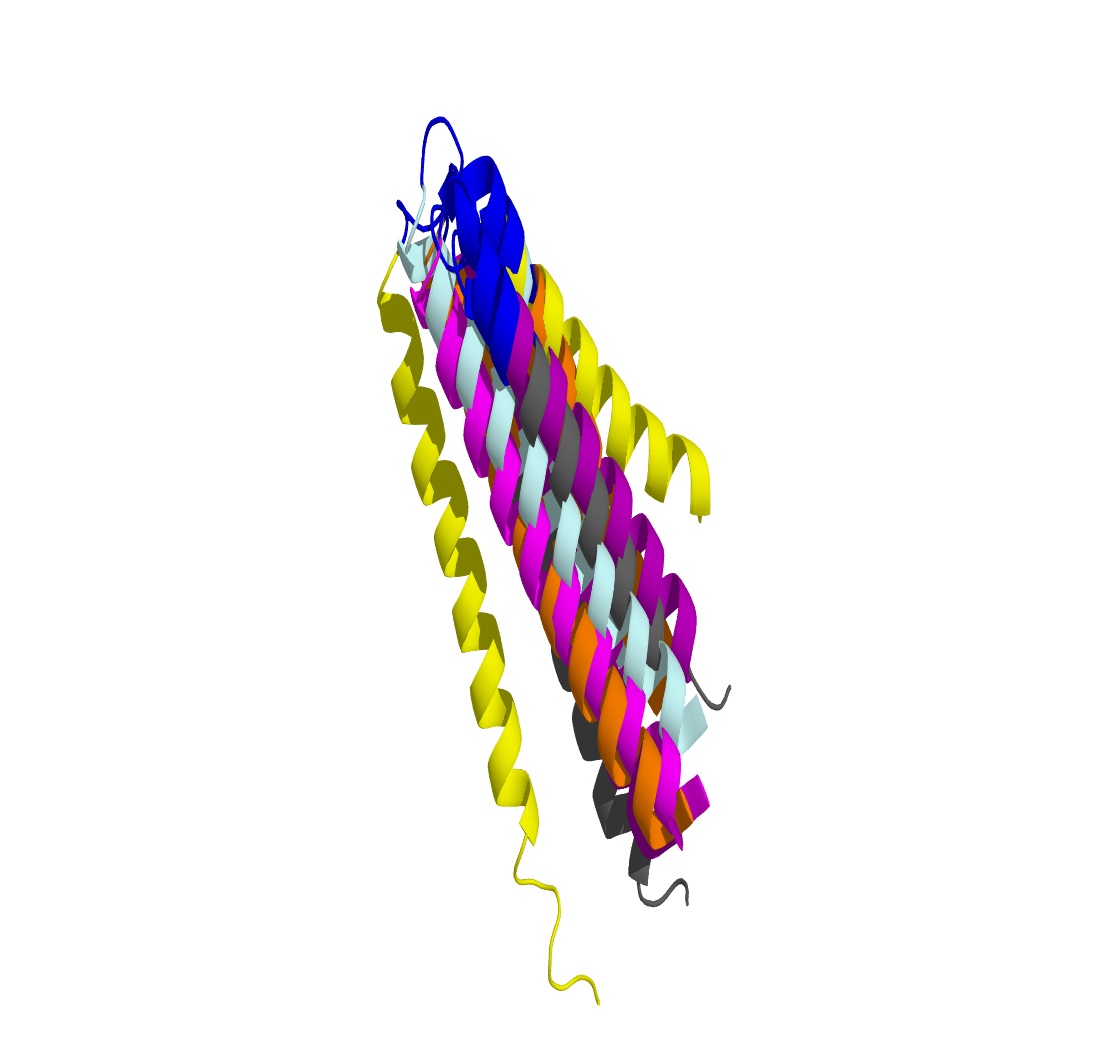

Supplement: Supplementary file 16 [file Image8.jpeg]
